# Effects of flowering period on floral traits, pollinator behavior and seed production of David’s mountain laurel (*Sophora davidii*)

**DOI:** 10.1080/15592324.2024.2383823

**Published:** 2024-07-27

**Authors:** Chengtao Pan, Zhimin Chen, Mao Zhang, Xiangsheng Chen, Guy Smagghe, Mingyu Fan, Zhimin Chang, Lili Zhao, Jiankun Long

**Affiliations:** a College of Animal Sciences, Guizhou University, Guiyang, China; b Institute of Entomology of Guizhou University, The Provincial Special Key Laboratory for Development and Utilization of Insect Resources of Guizhou University, Guiyang, China; c Department of Publicity, Donglan County Traditional Chinese Medicine Development Center, Hechi, China; d Department of Student, Guizhou Technological College of Machinery and Electricity, Duyun, China; e Department of Biology, Vrije Universiteit Brussel (VUB), Brussels, Belgium; f Department of Plants & Crops, Ghent University, Ghent, Belgium

**Keywords:** *Sophora davidii*, floral traits, flowering period, foraging behavior, pollination, yield, quality

## Abstract

*Sophora davidii* is a cross-pollinated plant with important ecological protection and medicinal value in China, but its seed yield is low due to backward and nonstandard production technology. Therefore, we divide the flowering period of *Sophora davidii* into initial, full and final flowering period, measuring the floral morphology, pollen viability, stigma receptivity, nectar volume and nectar concentration, foraging behavior of pollinators, fertilization physiology, seed yield and quality through field observation and indoor testing to explore whether the flowering period affects the floral traits, pollinator behavior and seed production of plants. Our results revealed that the nectar volume, nectar concentration, pollen viability and stigma receptivity at full flowering period were the highest. The single visit time and visit time per flower of Chinese honey bees were the longest in the full flowering period, while the number of transfer, visit frequency and number of touching stigma were the least. The visiting number of the bees was the most and the most active in the full flowering period. The bees pollination not only improved the pollen amount, germination rate, pollen tube length and the ovule number of *S. davidii*, but also their effect was the most obvious in full flowering period. The principal component analysis showed that pollination by Chinese honey bees during the whole flowering period of *S. davidii* was the best way to produce seeds. We can conclude that flowering period affects flower traits, foraging behavior of pollinators, seed yield and quality of *S. davidii*.

## Introduction

The David’s mountain laurel plant *Sophora davidii*, belongs to the Fabaceae family, is a spiny, deciduous, perennial shrub native to southwestern China, and typically grows from 2 to 4 m in length and width. It is not only an important pioneer plant for soil and water conservation and rocky desertification control in karst area of China, especially in the Guizhou province, but also a high-quality feed source for herbivorous livestock and one of the main nectar plants in spring.^1,2^ *S. davidii* is also used in the medical field. Its seeds and flowers contain a variety of alkaloids, flavonoids and other medicinal components, which can be used as effective components of therapeutic drugs for Alzheimer’s disease.^3^ Hence, its extract has certain inhibitory effects on Ebola virus and Lassa virus, and has various pharmacological activities such as anticancer, antitumor, antiviral, anti-inflammatory and anti-allergic.^4–6^

*S. davidii* is widely cultivated for its high ecological protection, forage grass and medicinal values, but its seed yield is low due to backward and unstandardized production technology.^7^ In addition, the successful fusion of male and female gametes in the process of seed formation depends on the factors such as pollinators, flower characteristics and various environmental conditions.^8–10^

*S. davidii* is a hermaphroditic plant that is cross-pollinated.^11^ Hence, in the absence of pollinators, the number of seeds per pod is small and even abortion exists.^12^ This means that the development and formation of the seeds of *S. davidii* is still dependent on the pollinators. However, studies on improving seed yield of *S. davidii* have focused mainly on cultivation and management, while studies on the pollination have been rarely reported.

Studies showed that about 76% of the world’s 115 major crops depended on bee pollination to form seeds and fruits.^13^ As pollinators, bees are able to pollinate in time, effectively, fully, recognized and safely, creating the necessary conditions for plant fertilization. Through bee pollination, more pollen cannot only fall on the stigma to germinate, making the group effect of pollen germination and pollen tube growth stronger, but also it can accelerate the physiological effect after fertilization and promote the development of fruits and seeds, thus achieving the effect of increasing production and improving quality.^14^ Among them, Chinese honey bees *Apis cerana cerana* Fabricius, as a native bee species in China, has the characteristics of diligent collection, strong resistance and adaptability, and is good at collecting sporadic nectar sources, which has been widely used in pollination of field crops and agricultural facility crops in China.

Pollination is a crucial step in the fertilization process of angiosperms, which means that active and mature pollen grains needs to be transported to the appropriate stigma by wind or pollinators such as bees.^15^ In the process of bee visiting flowers, the floral traits, flower reward and environmental factors will all affect their visiting behavior, and then affect their pollination effect. The success of pollination directly influences the success rate of plant reproduction.^16^ Because of this, it is necessary for us to observe the flower characteristics of plants and study pollination media in order to better understand the pollination methods of plants and lay a scientific foundation for choosing pollination methods.

However, the flower traits of plants vary with the growth cycle, and different floral traits also have certain effects on seed yield and quality.^17^ And the pollen viability and stigma receptivity of plants at different flowering periods are also different.^18^ It is evident that studying the flower characteristics of plants at different flowering periods is helpful to further understand the pollination and fertilization mechanism of plants, especially angiosperms.

Therefore, in order to explore the floral traits at different flowering periods and their effects on pollinator behavior and seed production, in this study, we measured the floral morphology, pollen viability, stigma receptivity, nectar volume and nectar concentration of *S. davidii* at different flowering periods, and in addition, we also investigated the foraging behavior of *A. cerana cerana* at different flowering periods and the effects of pollination treatment at different flowering periods on fertilization physiology, seed yield and quality of *S. davidii*. After collecting all the data, we will try to draw the correlation between flower traits and pollinator behavior, and determine the best pollination period of *S. davidii* through production performance, which will provide more meaningful reference value for rational pollination of insects, efficient production of seeds and honey production.

## Materials and methods

### Study site

The study area belongs to the subtropical monsoon climate and is located in the Guizhou Grassland Technology Test Extension Station (N25°04′~25°21′, E107°41′~107°55′), Dushan County, Guizhou Province, China. The lowest and highest temperature in the area was 2°C and 32°C, respectively, the annual precipitation about 1150 mm, and the annual sunshine hours about 1300 h.^19^

### Study materials

The main study was conducted from April to August 2021. The test material were 3-year-old potted *S. davidii* plants, which were transplanted to the test area upon reaching the flowering conditions. The pollinator was *A. cerana cerana* Fabricius, which is the locally dominant bee species in this study area.

## Experimental method

### Observation and determination of floral traits

The division of flowering periods was done according to the population flowering criteria as follows: (1) Initial is when 5% of the sample plants are flowering as the initial flowering period; (2) Full is when over 30% of the sample plants are flowering as the full flowering period; and (3) Final is when over 95% of the sample plants have been flowered as the final flowering period.^20^

We randomly marked 30 inflorescences from 30 plants of *S. davidii*, and observed their opening days from the first flower to the last flower. Another 30 flowers from different plants of *S. davidii* were randomly collected in the initial, full and final flowering periods, respectively. Each flowering periods was done with three repetitions, and each repetition had 10 flowers. The collected flower samples were placed in a centrifuge tube containing FAA fixative, marked and brought back to the laboratory for testing. The length of calyx, filament and pistil, and the length and width of corolla, anther, stigma, vexillum, wing and keel were measured with Vernier caliper with an accuracy of 0.02 mm.

### Nectar volume and nectar concentration

We randomly selected 10 inflorescences from 9 plants of *S. davidii* which isolated by net room at the initial, full and final flowering periods, respectively. The total nectar on each inflorescence was extracted by a 25-µL syringe, and the average nectar volume of each inflorescence was calculated. The nectar concentration was measured by a portable refractometer.

### Pollen viability and stigma receptivity

Another 30 flowers from 9 plants were randomly collected in three different periods as described above. The collected flower samples were placed in a centrifuge tube containing FAA fixative, marked and brought back to the laboratory, and then their pollens were dispersed by an oven at 45°C and stored in a refrigerator at 4°C for testing.

The pollen at the initial, full and final flowering periods of *S. davidii* were taken for histochemical evaluation. The pollen grains was distributed on slides and stained with I_2_-KI solution. We randomly selected three visual fields to observe the pollen staining. Blue is a well-developed and vigorous pollen grain, while yellow-brown is a poorly developed pollen grain. And the number of pollen grains in each visual field was not less than 100.^21^ The stigma receptivity was determined by the benzidine-hydrogen peroxide method^22^ with composition ratio 1% benzidine: 3% hydrogen peroxide: distilled water = 4:11:22.

### Foraging behavior of A. cerana cerana

Twenty seven neighboring *S. davidii* plants were randomly divided into nine plots (three plants per plot). At the initial flowering stage of *S. davidii*, three plots were randomly selected to observe the foraging behavior of *A. cerana cerana*, while the other six plots were covered with nylon nets (100 mesh of size). During the full flowering period, three of the remaining six plots were randomly selected as observation the foraging behavior of *A. cerana cerana*, while the remaining three plots and three plots at the initial flowering period were covered with the nylon nets. The foraging behavior of *A. cerana cerana* was observed in the remaining three plots at the final flowering period, while other plots were covered with nylon nets. The indices of foraging behavior was determined by visual inspection, stopwatch timing and camera shooting. All measurements were carried out on sunny and breezy days, and each flowering periods was repeated for 3 d as three repetitions. The definitions of indicators are as follows:

1. Single visit time: The time it takes for bees to collect nectar and pollen from flowers and leave the same flower.
2. Visit time per flower: The time it takes for bees to fall on flowers to collect nectar and pollen and temporarily leave flowers to comb pollen.
3. Whole visiting time: The time from the time when bees enter the pollination plot to collect nectar and pollen and leave the plot.
4. Number of transfer per minute: The transfer times of bees on flowers in the process of visiting flowers for 1 min.
5. Visit frequency: The number of flowers visited by bees within 1 min.
6. Number of touching stigma: The number of times that bees contact the style during the 1 min collection.
7. Number of visiting bee: The number of bees visiting a flower within 1 min in the pollination community.

### Pollination test

Five pollination treatments were set up, that is, bee pollination at initial flowering period (Initial pollination), bee pollination at full flowering period (Full pollination), bee pollination at final flowering period (Final pollination), bee pollination at whole flowering period (Whole pollination) and spontaneous self-pollination at whole flowering period (Self-pollination). Bee pollination treatment was to add *A. cerana cerana* colonies around the study area and let bees pollinate freely. However, spontaneous self-pollination in the whole flowering period uses the nylon net to cover the plants to avoid the influence of pollinators. Random block arrangement was used to carry out three replicates for each treatment, and three neighboring *S. davidii* plants were selected for each replication.

(1) Determination the indexes of fertilization physiology: 10 flowers pollinated by *A. cerana cerana* and 10 flowers pollinated without pollinators were randomly collected at the initial, full and final flowering period, respectively, and put into a centrifugal tube filled with FAA stationary solution and marked. After fixation for 24 h, it was washed with distilled water, and the pistil and ovary were taken out.

The pistils were softened with 8 mol/L NaOH solution and stained with 0.1% aniline blue solution to make slides. The indexes of fertilization physiology which included the amounts of pollen and germinated pollen on the stigma, the numbers of ovules in the ovary and the lengths of pollen tubes, were measured with use of an optical or fluorescence microscope.

(2) Determination of seed yield: The seed yield of *S. davidii* was evaluated by pod setting rate, seed setting rate and 1000-grain-weight. After pollination, 10 inflorescences were randomly marked in each treatment, and the pod setting situation was counted in the pod setting period. After pods matured, 10 pods were randomly collected in each treatment, and the seed setting situation of each pod was counted. 100 seeds were randomly selected from the seeds obtained after pollination, then weighed and converted into 1000-grain-weight. The counting unit was grams. Each treatment was repeated three times.

(3) Determination of seed quality: Seed quality was evaluated by seed germination test. Fifty seeds with full grains were selected for each treatment. The seeds were evenly placed in a Petri dish covered with filter paper, and placed in a constant temperature (25℃) and humidity (80%) incubator to observe and record the germination of seeds, the length of root and seedlings. Each treatment was repeated three times. The calculation formulas of seed germination rate, germination potential, germination index and total activity were as follows:

Germination rate (%) = (Number of seeds germinated during two weeks/Total number of seeds tested) × 100%

Germination potential (%) = (Number of germinated seeds when the seeds germinate to the peak of the day/Total number of tested seeds) × 100%

Germination index (GI) = ∑ *Gt*/*Dt*, (*Gt* is the number of seeds germinated in *t* days, and *D*t is the corresponding number of seeds germinated)

Vigor index (VI) = GI × *S* (‘GI’ is the germination index, and ‘*S*’ is the sum of the finally measured root length and seedling length)

### Statistical analysis

The software used for the collation, analysis and drawing of test data were Excel 2016, GraphPad Prism 9 and SPSS 27.0. Most of the data obeyed a normal distribution, while percentage data and nectar volume were transformed by $\arcsin\sqrt{x/100}$ and $\sqrt{x+0.5}$, respectively. One-way analysis of variance was used to compare the differences in floral morphology, pollen viability, stigma receptivity, nectar volume and nectar concentration at different flowering periods as well as to compare the effects of different pollination treatments on the seed yield and quality of *S. davidii*, and the two PC values were extracted by principal component analysis (PCA) to calculate the scores of different pollination treatments and to derive the optimal pollination method. The independent samples *t*-test was used to compare the changes of indexes of fertilization physiology in *S. davidii* after bee pollination and spontaneous self-pollination at different flowering periods.

## Results

### Floral traits

The phenological periods of *S. davidii* was 9.30 ± 0.24 d in the Guizhou Grassland Technology Extension Station of Dushan County, Guizhou Province, China. The initial, full and final flowering periods were 3.00 ± 0.14 d, 3.43 ± 0.13 d and 2.93 ± 0.16 d, respectively.

As shown in Table 1, there was no significant difference in calyx length, anther length, anther width, stigma length and stigma width during the three flowering periods (*p* > .05). But, the indices of stamen length, corolla length, corolla width, wing length, wing width and keel length at full flowering period were significantly lower than those at initial flowering period and final flowering period (*p* < .05). Among the three flowering periods, the pistil length at the full flowering period was only significantly longer than the length at the initial flowering period (*p* < .05). The vexillum length only showed that the length at full flowering period was significantly longer than the length at initial flowering period and the final flowering period (*p* < .05). The order of vexillum widths were as follows: initial flowering period > final flowering period > full flowering period (*p* < .05). The keel width at the initial flowering period, was only significantly wider than the width at the full flowering period (*p* < .05).

**Table 1.** The floral morphology of *S. davidii* at different flowering periods.

Parameter (mm)	Initial	Full	Final
Calyx length	5.13 ± 0.27a	5.90 ± 0.27a	5.35 ± 0.18a
Stamen length	9.34 ± 0.71a	6.57 ± 0.17b	8.79 ± 0.06a
Pistil length	6.13 ± 0.34b	8.94 ± 0.26a	7.55 ± 1.19ab
Corolla length	8.95 ± 1.82a	3.85 ± 0.56b	8.80 ± 1.46a
Corolla width	4.71 ± 0.50a	1.75 ± 0.21b	4.17 ± 0.34a
Anther length	1.14 ± 0.13a	1.08 ± 0.05a	1.12 ± 0.01a
Anther width	0.69 ± 0.10a	0.72 ± 0.03a	0.95 ± 0.15a
Stigma length	0.16 ± 0.01a	0.15 ± 0.01a	0.17 ± 0.01a
Stigma width	0.15 ± 0.01a	0.15 ± 0.01a	0.15 ± 0.00a
Vexillum length	7.55 ± 0.33b	9.96 ± 0.62a	7.64 ± 0.50b
Vexillum width	4.70 ± 0.14a	2.85 ± 0.14c	3.94 ± 0.23b
Wing length	13.70 ± 0.26a	11.00 ± 0.31b	13.34 ± 0.13a
Wing width	4.36 ± 0.20a	2.97 ± 0.09b	4.32 ± 0.16a
Keel length	12.28 ± 0.59a	10.01 ± 0.49b	12.57 ± 0.21a
Keel width	4.15 ± 0.41a	2.86 ± 0.11b	3.42 ± 0.16ab

The values in the table are “mean ± SE”, based on three repetitions and each repetition consisted of 10 flowers per flowering stage. Different letters represent significant differences in different flowering periods (*p* < .05).

**Figure 1.**
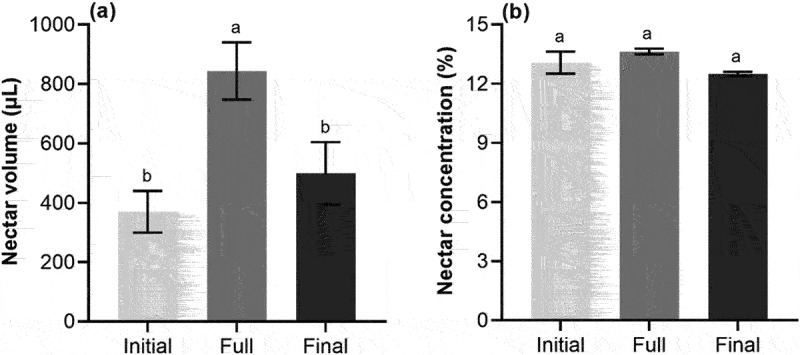
Mean (±SE) nectar volume (a) and nectar concentration (b) from *S. davidii* at different flowering periods. Based on three repetitions and each repetition consists of 30 inflorescences per flowering period. Different letters indicate a significant difference in different flowering periods (*p* < .05).

There were significant differences in nectar volume in different flowering periods (*p* < .05; Figure 1a), but no significant difference in nectar concentration (*p* >.05; Figure 1b). Among them, the maximum volume of nectar is 844 ± 11 μL at full flowering period, followed by 499 ± 2 μL at final flowering period and 370 ± 6 μL at initial flowering period. Although there was no difference in nectar concentration, the highest nectar concentration was 13.6 ± 0.2% at full period.

There were significant differences in pollen viability of *S. davidii* in different flowering periods (Table 2). The results of the I_2_-KI staining showed that the pollen viability at full flowering period was significantly higher than that at initial and final flowering period. Although the pollen viability at initial flowering period was higher than that at final flowering period, the difference was not significant (*p* > .05).

**Table 2.** Pollen viability (mean ± SE) and stigma receptivity of *S. davidii* at different flowering periods, initial, full and final.

Periods	Initial	Full	Final
Pollen viability (%)	27.33 ± 5.72b	75.00 ± 5.24a	20.56 ± 0.29b
Stigma receptivity	++	+++	+

Based on three repetitions and each repetition consists of 30 flowers per flowering period. Different letters represent significant differences in different flowering periods (*p* < .05). “+++” indicates strong receptivity. “++” indicates low receptivity. “+” indicates very low receptivity.

The stigma of *S. davidii* had a receptivity at each flowering period, but the strongest was at full flowering period (*p* < .05). It was thus clear that *S. davidii* had high pollen viability and stigma receptivity at full flowering period.

### Foraging behavior

**Figure 2.**
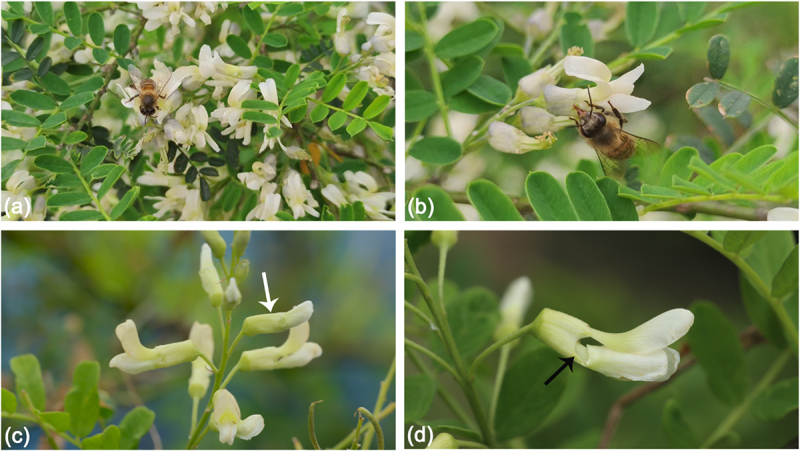
Foraging behavior of *A. cerana cerana* on *S. davidii*. (a) Pollen foraging behavior, (b) nectar foraging behavior, (c) flowers that have not been foraged, (d) flowers after foraging. (white arrow: undamaged. Black arrow: damaged.).

The nectar foraging behavior and pollen foraging behavior of *A. cerana cerana* on *S. davidii* are shown in Figure 2. Because the head and beak of *A. cerana cerana* cannot suck nectar from the flower base, it is observed that they will bite an opening near the flower base 2/5 (Figure 2d), and this nectar robbing behavior happens in most flowers.

**Figure 3.**
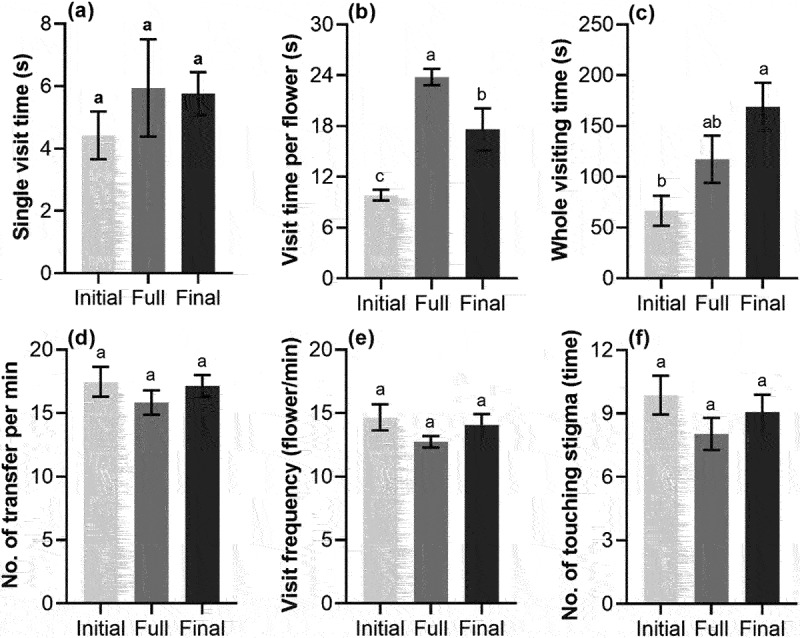
Foraging behavior (mean ± SE) of *A. cerana cerana* at different flowering periods of *S. davidii*, initial, full and final. (a) Single visit time, (b) visit time per flower, (c) whole visiting time, (d) number of transfer per minute, (e) visit frequency, (f) number of touching stigma. Different letters indicate a significant difference in different flowering periods (*p* < .05).

The foraging behavior of *A. cerana cerana* at different flowering periods of *S. davidii* is shown in Figure 3, in which the visit time per flower at full flowering period was significantly more than that at initial flowering period and final flowering period (*p* < .05), and the time at final flowering period was also significantly more than that at initial flowering period (*p* < .05). The whole visiting time at the final flowering period was significantly more than that at the initial flowering period (*p* < .05). However, there was no significant difference in the single visit time, the number of transfer per minute, the visit frequency and the number of touching stigma at different flower periods (*p* > .05).

**Figure 4.**
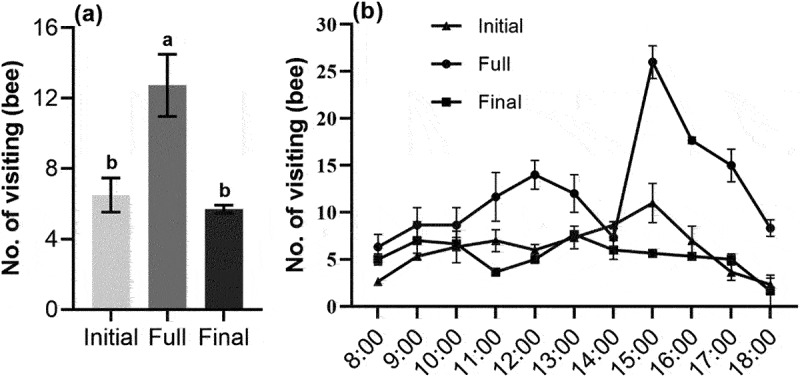
The number and dynamics of visiting bees (mean ± SE) at different flowering stages of *S. davidii*, initial, full and final. (a) Number of visiting bees, (b) the dynamic of visiting bees. Different letters indicate a significant difference in different flowering periods (*p* < .05).

The number of bees visiting flowers in the full flowering period of *S. davidii* was significantly higher than that at the initial flowering period and the final flowering period (*p* < .05) (Figure 4). Although the dynamic changes of the number of bees at different flowering periods were different, they all showed a “double peak” change. Such as the initial flowering period (11:00 and 13:00), the full flowering period (12:00 and 15:00), and the final flowering period (9:00 and 13:00), as well as the number of bees increased sharply at 15:00 in the full flowering period.

According to the results of correlation analysis (Table 3), there was no significant correlation between the single visit time and total visit time of Chinese honey bees and the floral traits of *S. davidii* (*p* > .05), but the visit time per flower, transfer times, visit frequency, times of touching stigma and number of visits were significantly (*p* < .05) or extremely significantly (*p* < .01) related to the flower traits.

**Table 3.** Correlation between floral traits of *S. davidii* and foraging behavior.

Parameter	SVT	VTF	WVT	NTM	VF	NTS	NVB
CAL	−0.442	0.652	0.069	−0.822**	−.871**	−0.789*	0.616
SAL	−0.264	−0.794*	−0.206	0.651	0.548	0.671*	−0.885**
PL	0.022	0.689*	0.360	−0.851**	−0.947**	−0.743*	0.607
CL	−0.014	−0.668*	−0.079	0.648	0.635	0.627	−0.786*
CW	−0.355	−0.819**	−0.210	0.647	0.659	0.714*	−0.887**
AL	−0.414	−0.207	−0.226	−0.003	−0.207	−0.072	−0.261
AW	0.073	0.081	0.553	−0.225	−0.409	−0.095	−0.330
SIL	0.002	−0.316	0.160	0.153	0.162	0.028	−0.618
SIW	−0.301	−0.178	−0.058	−0.028	−0.110	−0.023	−0.290
VL	−0.071	0.630	−0.121	−0.777*	−0.724*	−0.922**	0.785*
VW	−0.073	−0.950**	−0.334	0.610	0.648	0.689*	−0.810**
WL	−0.240	−0.823**	−0.143	0.684*	0.645	0.698*	−0.941**
WW	0.158	−0.774*	0.071	0.703*	0.656	0.784*	−0.926**
KL	−0.098	−0.626	0.070	0.513	0.428	0.552	−0.918**
KW	−0.143	−0.813**	−0.441	0.583	0.443	0.569	−0.669*
NV	−0.010	0.837**	0.223	−0.553	−0.659	−0.592	0.791*
NC	−0.323	0.298	−0.367	−0.344	−0.509	−0.394	0.623
PV	−0.165	0.499	−0.419	−0.503	−0.563	−0.627	0.923**
SR	0.060	0.658	−0.213	−0.521	−0.534	−0.640	0.952**

Calyx length=CAL, Stamen length =SAL, Pistil length=PL, Corolla length=CL, Corolla width=CW, Anther length=AL, Anther width=AW, Stigma length=SIL, Stigma width=SIW, Vexillum length=VL, Vexillum width=VW, Wing length=WL, Wing width=WW, Keel length=KL, Keel width=KW, Nectar volume=NV, Nectar concentration=NC, Pollen viability=PV, Stigma receptivity=SR, Single visit time=SVT, Visit time per flower=VTF, Whole visiting time=WVT, Number of transfer per minute=NTM, Visit frequency=VF, Number of touching stigma=NTS, Number of visiting=NVB. * and ** indicate significant correlation at 0.05 and 0.01 levels, respectively.

### Fertilization physiology

**Figure 5.**
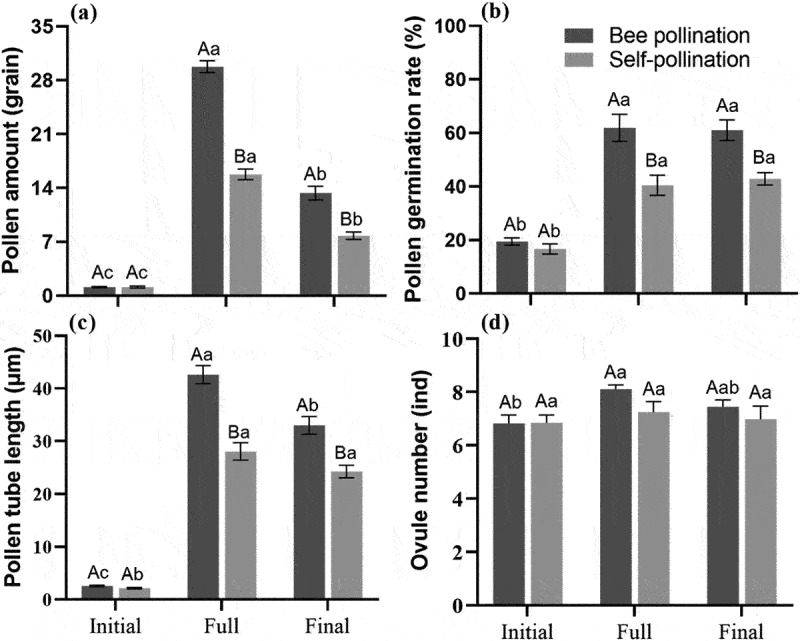
Mean (± SE) fertilization physiology indices at different flowering periods in *S. davidii* after pollination. (a) pollen amount, (b) pollen germination rate, (c) pollen tube length, (d) ovule number. Different lowercase letters indicate a significant differences in different flowering periods (*p* < .05), and different uppercase letters indicate a significant differences between different pollination methods in the same flowering period (*p* < .05).

There were significant differences in pollen amount on, pollen germination rate and pollen tube length during different flowering periods under the two pollination methods (Figure 5; *p* < .05), and also some differences under different pollination methods at the same flowering period. In terms of stigma pollen amount (Figure 5a), the significance of bee pollination and self-pollination during the three flowering periods was as follows: full flowering period > final flowering period > initial flowering period (*p* < 0.05). Bee pollination scored significantly higher than self-pollination at full flowering period (F = 0.083, *t* = 13.587, *p* < 0.05) and final flowering period (F = 1.225, *t* = 5.522, *p* < .05). In terms of pollen germination rate (Figure 5b), both bee pollination and self-pollination showed that the full and final flowering period were significantly higher than the initial flowering period (*p* < .05), and bee pollination was significantly higher than self-pollination at the full (F = 2.838, *t* = 3.909, *p* < .05) and final flowering period (F = 1.643, *t* = 4.460, *p* < .05). In terms of pollen tube length (Figure 5c), the significance of bee pollination during the three flowering periods was as follows: full flowering period > final flowering period > initial flowering period (*p* < .05). While self-pollination showed that the full and final flowering period were significantly greater than the initial flowering period (*p* < .05), but there was no significant difference between full flowering period and final flowering period (*p* > .05). Bee pollination was significantly greater than self-pollination at full flowering period (F = 0.001, *t* = 6.149, *p* < .05) and final flowering period (F = 0.256, *t* = 4.235, *p* < .05). On the number of ovules (Figure 5d), the full flowering period was significantly greater than the initial flowering period only in the presence of the Asian honey bees that were pollinating (*p* < .05).

### Seed yield

Different pollination treatments have significant effects on pod setting rate, seed setting rate and thousand-grain weight of *S. davidii* (Table 4). Among them, the pod setting rate at full flowering period was the highest, the rate of which was significantly higher than other pollination treatments (*p* < .05). The seed setting rate in full flowering period, was also the highest, which was significantly higher than other pollination treatments except full flowering period (*p* < .05). However, the highest thousand-grain weight of seeds was the whole pollination treatment, which was significantly heavier than both treatments of the final and self-pollination (*p* < .05).

**Table 4.** Effects of different pollination treatments on seed yield of *S. davidii.*

Pollination treatment	Pod setting rate (%)	Seed setting rate (%)	1000 - grain weight (g)
Initial pollination	50.33 ± 1.26b	47.67 ± 4.10b	2.15 ± 0.07ab
Full pollination	64.20 ± 2.32a	59.67 ± 4.33a	2.09 ± 0.04abc
Final pollination	7.20 ± 1.11d	32.67 ± 3.84c	2.05 ± 0.02bc
Whole pollination	37.87 ± 1.99c	58.33 ± 2.34ab	2.26 ± 0.01a
Self-pollination	7.33 ± 0.84d	30.67 ± 1.86c	1.89 ± 0.11c

The values in the table are “mean ± SE”, and different lowercase letters in the same column indicate significant differences (*p* < .05).

### Seed quality

The results in Table 5 show that different pollination treatments had significant effects on the seed quality of *S. davidii*. Among them, the germination rate of whole flowering pollination was the highest, followed by full flowering pollination, and the final flowering pollination was the lowest. The germination rates of whole flowering pollination and full flowering pollination were significantly higher than other pollination treatments (*p* < .05). The highest germination potential was whole flowering pollination which was significantly higher than that of other treatments except full flowering pollination (*p* < .05). And the highest germination index was full flowering pollination which was significantly higher than other pollination treatments (*p* < .05). However, the highest index of vigor was full flowering pollination, followed by initial flowering pollination, but only the vigor index of full flowering pollination and initial flowering pollination were significantly higher than that of self-pollination (*p* < .05).

**Table 5.** Effects of different pollination treatments on seed quality of *S. davidii.*

Pollination treatment	Germination rate (%)	Germination potential (%)	Germination index	Vigor index
Initial pollination	74.67 ± 2.67b	25.83 ± 1.17b	8.18 ± 0.79b	212.45 ± 40.81a
Full pollination	84.81 ± 1.85a	32.43 ± 1.23ab	10.87 ± 0.14a	242.24 ± 92.35a
Final pollination	64.22 ± 5.25c	24.47 ± 2.23b	5.82 ± 0.28c	119.10 ± 4.70ab
Whole pollination	91.33 ± 1.02a	39.10 ± 7.19a	6.28 ± 0.22c	147.87 ± 58.08ab
Self-pollination	72.67 ± 0.66bc	13.33 ± 0.19c	2.13 ± 0.06d	26.71 ± 1.09b

The values in the table are “mean ± SE”, and different lowercase letters in the same column indicate significant differences (*p* < .05).

### Principal component analysis (PCA)

PCA shows that (Table 6) there were two PC values greater than 1, and their cumulative contribution rate reached 91.36% (76.94% and 14.42%, respectively), which could be used as a comprehensive evaluation index of *S. davidii* seed yield and quality under different pollination treatments. The principal components, extracted in Table 4, were converted into normalized feature vectors. And the score calculation model and comprehensive evaluation model of the two principal components obtained as follows:

**Table 6.** Principal component extraction of seed yield and quality of *S. davidii* after different pollination treatments.

Parameter	Component matrix	
	Principal component 1	Principal component 2
Pod setting rate	0.911	−0.286
Seed setting rate	0.976	0.134
Thousand-grain weight	0.828	0.325
Germination rate	0.768	0.508
Germination potential	0.896	0.324
Germination index	0.861	−0.487
Vigor index	0.885	−0.452
Eigenvalue	5.386	1.010
Variance contribution rate	76.936	14.424
Cumulative contribution rate	76.936	91.360

Y_1_ = 0.393X_1_ +0.420X_2_ +0.357X_3_ +0.331X_4_ +0.386X_5_ +0.371X_6_ +0.381 X_7_;

Y_2_ = −0.285 X_1_ +0.134 X_2_ +0.324X_3_ +0.505X_4_ +0.322X_5_ −0.485X_6_ −0.450X_7_;

Y = 0.7694Y_1_ +0.1442Y_2_。

In the formula, X_1_=Pod setting rate，X_2_=Seed setting rate，X_3_=Thousand-Grain Weight，X_4_=Germination rate，X_5_=Germination potential，X_6_=Germination index，X_7_=Vigor index.

The higher the comprehensive score, the more favorable pollination treatment was to increase the yield and improve the quality of *S. davidii* seeds. It could be seen from Table 7 that the comprehensive score of pollination at full flowering stage was the highest (2.83), followed by pollination at full flowering stage (0.99), and self-pollination was the lowest (−2.14).

**Table 7.** Principal component factor scores of seed yield and quality of *S. davidii* after different pollination treatments.

Pollination treatment	Y_1_ (Ranking)	Y_2_ (Ranking)	Y (Comprehensive ranking)
Initial pollination	0.80 (3)	−0.76 (4)	−0.08 (3)
Full pollination	2.25 (1)	−0.81 (5)	0.99 (2)
Final pollination	−1.65 (4)	−0.36 (3)	−1.60 (4)
Whole pollination	1.76 (2)	1.61 (1)	2.83 (1)
Self-pollination	−3.16 (5)	0.32 (2)	−2.14 (5)

## Discussion

Floral traits and flowering phenology of plants are important for studying the reproductive ecology of plants. Among them, it is accepted that the flowering time and duration, flower structure characteristics and nectar volume have a significant effect on the plant reproductive success and pollinator behavior.^10,23^ The flowering periods of *S. davidii* population are happening between March and May, and the flowering periods of a single flower take about 9 d. The times of initial, full and final flowering periods were all a duration of about 3 d The flowering period of a single flower is consistent with the description of the Apicultural Science Association of China.^24^

The pistil of *S. davidii* is wrapped in the stamens. The pistil is short and the stamens are long at the initial and final flowering period, while the full flowering period is the opposite. This is similar to some of the interactive dimorphic stamens in family Rubiaceae, which is beneficial for insects to achieve accurate pollination and improve mating efficiency.^25^ In addition, nectar volume of *S. davidii* was abundant, and the sugar concentration of nectar was higher (about 13%). The secretion of nectar began at the initial flowering period, while the most vigorous was at the full flowering period, and there was still a high amount of nectar volume at the final flowering period. The nectar volume at the full flowering period was significantly greater than that at the initial and final flowering periods, which could provide a rich nectar reward for pollination insects throughout the phenological period of *S. davidii*. The result was similar to the nectar secretion rhythm of the new branches of *Lycium barbarum*, also known as Chinese wolfberry in China or Duke of Argyll’s tea tree in the UK, that is an important commercial shrub crop in northern China.^26^ But, *S. davidii* has a peak honey production period of about 20 d, and throughout all the flowering periods it can brew between 20 and 25 kg of first-class honey per colony of bees,^24^ which is reflecting that *S. davidii* is an ideal plant of nectar source and honey production.

Pollen, as the male gametophyte of plants, plays an important role in sexual reproduction, and active pollen can germinate on the stigma to complete fertilization.^27^ But, the pollen viability of *S. davidii* was changing over the different flowering periods and was as follows: full > initial > final flowering period (Table 2), and we believe this may be caused by plant genetic genes or environmental conditions as was also recently reported by Karim et al.^28^

The period of stigma receptivity is the reproductive period in the process of flower maturation, which can affect to a large extent the self-pollination rate and the pollination rate at different periods after flowering. For example, the stigma receptivity of *G. rigescens* is weak at the end of the bud, strong at the full flowering period, and strongest at the late of the flowering period, but there is no receptivity at the wilting period. This is in agreement with previous work that the stigma receptivity of flowering plants is different at different opening periods of flowers.^29,30^ The results of this study showed that the stigma of *S. davidii* had a strong receptivity at full flowering period, and some stigmas had a receptivity at the initial and final flowering period (Table 2), indicating that *S. davidii* was suitable for pollination during the whole phenophase, but its pollen viability and stigma receptivity were the best at full flowering period.

The collection behavior of bees on pollinating plants is usually used as an evaluation index of bee pollination ability and effectiveness, which is generally influenced by plant resources, environmental factors and their own physiological factors.^31,32^ It is a mutually beneficial process for bees to visit flowering plants. Plants can provide some rewards for bees, such as nectar and pollen, which are stored in the hive as the energy source for their reproduction. At the same time, the collection behavior of bees increases the chances of flower pollination and improves the cross-pollination rate.^25,33^ However, due to the limited resources and the change of flower morphology, there are some differences in the behavior of bees in different collection periods. In this study, the visit time (such as single visit time, visit time per flower and whole visiting time) of bees in the initial flowering period was less than that in the full flowering period and the final flowering period, so bees needed to move and visit between different flowers frequently, which led to higher transfer times, visit times and times of touching the stigma (Figure 2). However, there were many flowers and available resources in the full flowering period, so more time was spent on a flower, which led to the least number of transfer per minute, visit frequency and number of touching stigma in the full flowering periods, but it was not statistically significant among the three flowering periods (Figure 2). Some researchers believe that the most effective pollinators should be large in number and move quickly between flowers.^34^ In this study, the bees in full flowering period were the most active, indicating that the full flowering period was an effective period for Chinese honey bees to pollinate *S. davidii*.

Bee pollination can bring more pollen to the plant stigma, promote pollen germination on the stigma and provide a strong guarantee for the germination of more pollen tubes and double fertilization on the stigma.^35^ The results of this study demonstrated that the pollen amount and germination rate of *S. davidii* pollinated by the local bees (*A. cerana cerana*) was higher than those of self-pollination (Figure 4). It might be affected by bee pollination, which increases the probability of cross-pollination of *S. davidii* and promotes pollen germination.^36,37^ When comparing bee pollination and self-pollination (Figure 4), the amount of pollen at the initial flowering period were the same, which may be related to the volatile substances of flowers and the frequency of bee visits.^38,39^

Similar to the pump pollination mechanism of family Papilionidae, the self-pollination efficiency of *S. davidii* is low, and it depends on strong and specialized pollinators.^40–42^ In this study, compared with self-pollination at whole flowering period, the pod setting rate, seed setting rate, thousand-grain weight, germination rate, germination potential, germination index and vigor index of bee pollination at whole flowering period increased by 30.54%, 27.66%, 0.37 g, 18.66%, 25.77%, 4.15 and 121.16, respectively (Tables 4 and 5). This increase could be due to frequent foraging activities caused by the introduction of bees which reduces the pollination failure and less number of flowers left without proper pollination.^43^ From the perspective of pollinator’s body shape, *A. cerana cerana* may not be the best pollinator of *S. davidii*, but can significantly improve the seed yield and quality of *S. davidii*. And pollination by *A. cerana cerana* during the whole flowering period may be the best way to promote its seed production.
